# Review detection of Newcastle disease virus

**DOI:** 10.3389/fvets.2022.936251

**Published:** 2022-08-02

**Authors:** Qian Mao, Shengming Ma, Philip Luke Schrickel, Pengwei Zhao, Jingya Wang, Yuhua Zhang, Shuangyu Li, Chengbao Wang

**Affiliations:** ^1^College of Veterinary Medicine, Northwest Agriculture and Forestry University, Xianyang, China; ^2^Henan Joint International Research Laboratory of Veterinary Biologics Research and Application, Anyang Institute of Technology, Anyang, China

**Keywords:** molecular assay, serological assays, Newcastle disease virus, historical overview, detection strategies

## Abstract

Newcastle disease (ND) is an acute and highly contagious disease caused by the Newcastle disease virus (NDV) infecting poultry, which has caused great harm to the poultry industry around the world. Rapid diagnosis of NDV is important to early treatment and early institution of control measures. In this review, we comprehensively summarize the most recent research into NDV, including historical overview, molecular structure, and infection mechanism. We then focus on detection strategies for NDV, including virus isolation, serological assays (such as hemagglutination and hemagglutination-inhibition tests, enzyme linked immunosorbent assay, reporter virus neutralization test, Immunofluorescence assay, and Immune colloidal gold technique), molecular assays (such as reverse transcription polymerase chain reaction, real-time quantitative PCR, and loop-mediated isothermal amplification) and other assays. The performance of the different serological and molecular biology assays currently available was also analyzed. To conclude, we examine the limitations of currently available strategies for the detection of NDV to lay the groundwork for new detection assays.

## Introduction

Newcastle disease (ND), which is caused by infections of poultry species with virulent strains of Newcastle disease virus (NDV), also known as avian paramyxovirus 1 (APMV-1), may cause acute, virulent, and highly contagious infectious diseases of poultry, characterized by damage to the digestive tract and central nervous system (1). It not only restricts the development of the poultry industry due to high morbidity and mortality rates caused by ND but also restricts the development of the poultry industry. According to reports, there have been at least four major outbreaks of ND around the world, which have caused great harm to international trade, and each epidemic had its specific genotype (2). Despite the wide use of ND vaccination programs in the poultry industry, different wild strains of the immunized flock are still isolated (3). Simultaneously, the emergence of atypical clinical symptoms and new strains has posed many difficulties in diagnosis, prevention, and control of the disease. With the development of the global livestock and poultry industry and the increase in world trade, the prevention of ND is particularly important. Early detection *via* rapid, sensitive, and accurate methods is very important for reducing the spread of ND (4). This review introduces the latest research on ND, including historical overview, structural biology, and infection mechanism. In addition, there are comprehensive summary detection strategies for ND, including viral isolation, serological assays, and molecular assays.

### Historical overview

**Figure 1:**
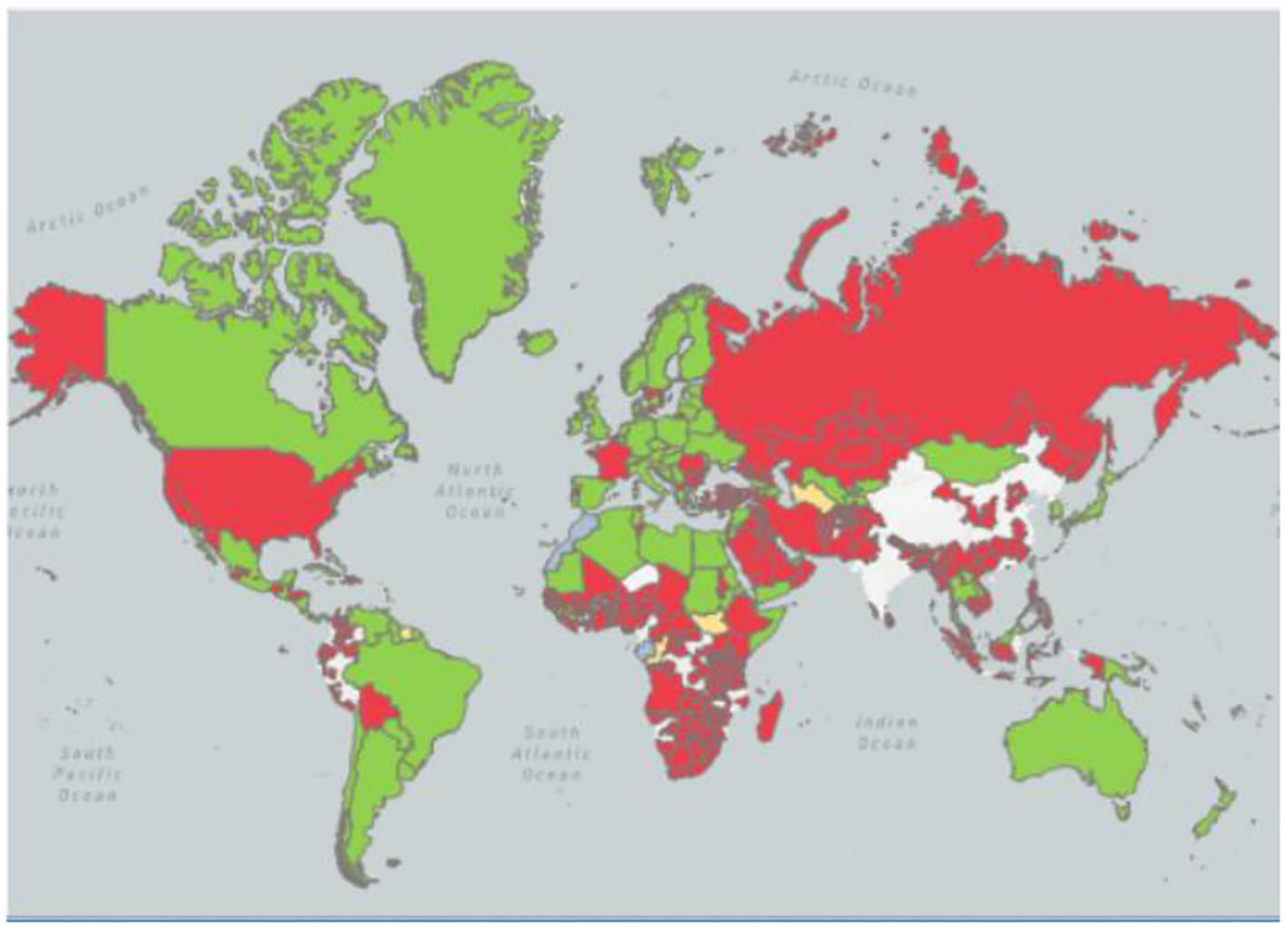
The prevalence of OIE in NDV in the past 5 years.

In 1926, ND was first reported to Indonesia (5, 6). In 1927, Doyle isolated the virus in Newcastle, England, and named it NDV (7). Then numerous outbreaks of different avian species associated with virulent NDV were reported in North America (United States of America, Canada, and Costa Rica), Europe (France, Italy, England, Scotland, Spain, and Russia), Africa (Kenya), Asia (China, Korea, India, Japan, Sri Lanka, Saudi Kazakhstan, Philippines, and Arabia) and Australia (8, 9). Since 1926, there have been four ND pandemics worldwide, and each pandemic is caused by a different genotype of NDV. The first global pandemic, which began simultaneously in Southeast Asia and Europe from 1920 to 1960, took approximately 30 years to become fully established. It was caused by NDV of the three genotypes of genes II, III, and IV (10). The main infection targets are chickens, waterfowl, and birds, with almost no disease, showing certain regional distribution differences (11). The second pandemic from 1960 to 1970 may have originated in the Middle East due to the increased commercialization of the poultry industry globally, as well as the enhanced international trade of parrots. This was mainly caused by V and VI genotypes of NDV, which endanger ornamental birds and caged birds (12, 13). The third pandemic believed to be caused by genotype VI isolates from late 1970 to 1980 occurred among racing pigeons but then spread all over the world and became difficult to control due to the relative lack of absolute control in racing pigeons' husbandry (14). Finally, the fourth pandemic which is currently ongoing is believed to have started in the late 1980s and has been related to genotypes VII, V, VI, and VIII. It has caused economic losses in the poultry industry in many countries in Southeast Asia, the Middle East, Europe, Africa, and the Americas (15–17) (Figure 1).

### Structural biology of NDV

**Figure 2:**
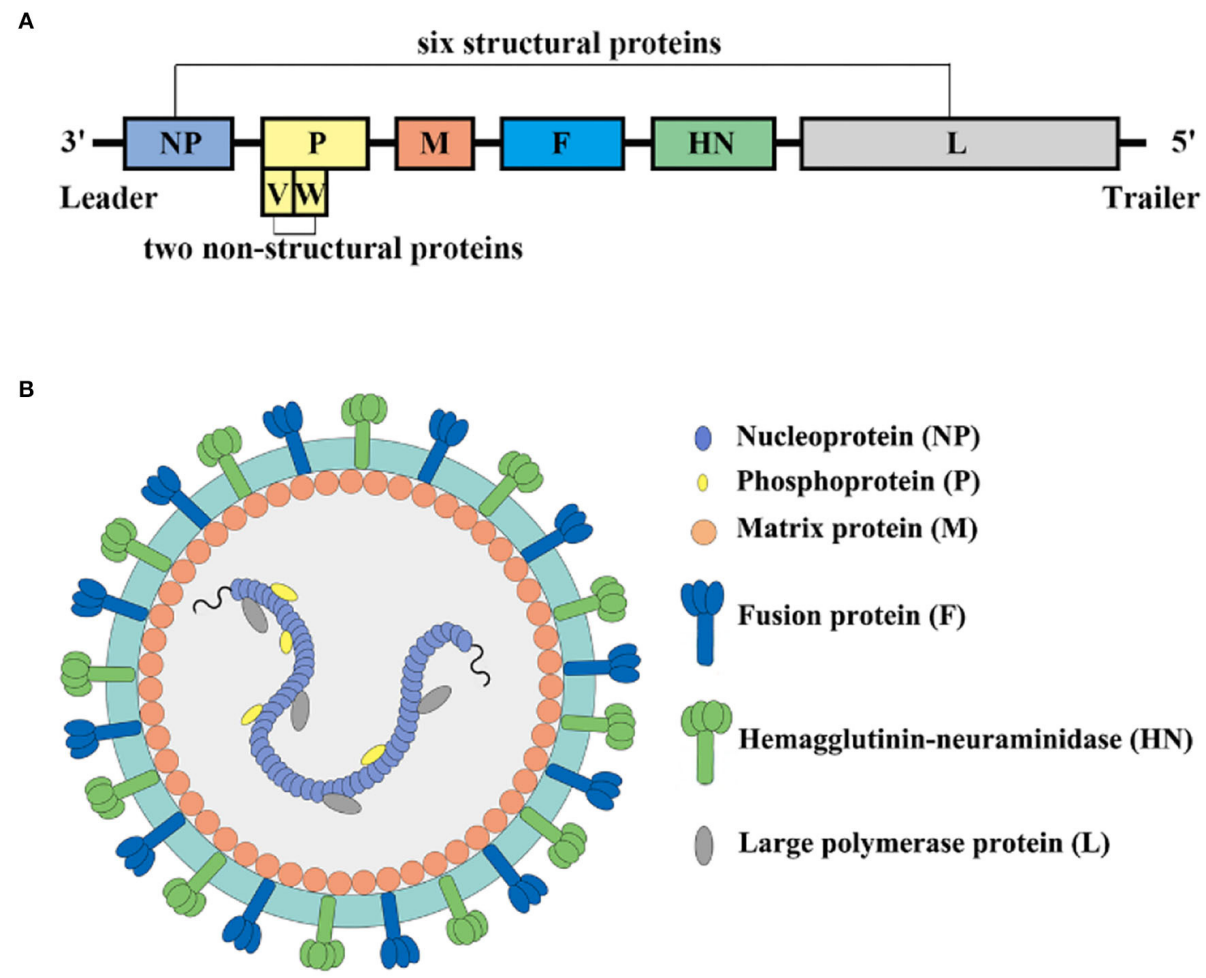
**(A)** Schematic diagram of NDV genome; **(B)** Schematic diagram of NDV structure.

NDV is a non-segmented, negative-sense, single stranded RNA virus in the family Paramyxoviridae that consists of leader (55 nucleotides) and trailer (114 nucleotides) terminal sequences separated, with a full-length genome of 15192, 15198, or 15186 bp (18, 19). In terms of ultrastructure, the particles of NDV range from 100 to 250 nm in diameter with spherical (most common) and filamentous forms. The core of the NDV virus particle is composed of a helical symmetrical nucleocapsid, surrounded by an envelope, and the surface of the vesicle is covered with 8 nm long fibrils. NDV contains six structural proteins and two non-structural proteins in the order nucleoprotein (NP), phosphoprotein (P), matrix protein (M), fusion protein (F), hemagglutinin neuraminidase protein (HN), and large protein (L) (20, 21) (Figure 2).

The NP protein is the main structural protein of NDV nucleocapsid protein, and it is also the most abundant protein present in the viral particles (22). The NP-protein forms the helical nucleocapsid complex about the viral genomic RNA, which protects the viral genomic RNA from RNase degradation and is important to genome packaging into mature virus particles. NM2-1 protein is a transcriptional anti-termination factor, which combines with NP protein, P protein, L protein, and viral genomic RNA to form the RNP complex as a template for RNA replication and transcription (23).

The P and L protein form a P-L complex and play a vital role in the replication and transcription of NDV. The P-L complex replicates the genome and synthesizes full-length plus-strand antigenomic RNA, which serves as a template for minus-strand genomic RNA (23). The P protein helps stabilize the L protein in the P-L complex and acts as a viral RNA-dependent RNA polymerase (vRdRp) (24, 25). The N protein wraps viral RNA to form the N-RNA template as the nucleocapsid core (NC), the RNA polymerase is composed of the L protein and the cofactor P protein combined with the N-RNA template to form the active ribonucleoprotein (RNP) complex required for transcription and replication. The tetramer of phosphoprotein mediates the interaction between L protein and N-RNA template to prevent random encapsidation of non-viral RNA by N protein (26). In addition, during the nascent chain assembly step of genome replication, the P protein forms a complex with unassembled N protein which regulates the shift from transcription to replication. The P gene encodes two other non-structural proteins (V and W). The V protein, otherwise known as the antagonist protein, mediates the escape of viral IFN to escape the host's innate immune response (27, 28). The mechanism of W protein has to be investigated in the future.

The M protein is a non-glycosylated membrane-associated structural protein, which is located at the inner surface of the NDV envelope. It associates with the cytoplasmic domain of the F protein and constitutes the bridge between the viral envelope and the nucleocapsid (29). The M protein is demonstrated to be a nucleocytoplasmic shuttling protein with its nuclear localization sequences and therefore it does not require other NDV proteins to perform the nuclear localization function (30). The M protein undergoes nuclear-cytoplasmic shuttling through its nuclear localization signal during the early phases of virus infection, and these M proteins become ubiquitinated at their nuclear localization sequences. The nuclear localization of the M protein has the function of inhibiting host gene transcription and protein synthesis and ensuring the replication and transcription of the viral genome in the cytoplasm proceed (31). However, there is not yet fully understood about the nuclear localization functions of NDV M protein.

The F protein has significance as a type-I integral membrane protein and is the main determinant of NDV virulence. In infected cells, NDV-F is synthesized as a precursor F0 without fusion activity, and then it is activated *via* cleavage by host protease of the Fc into two disulfide-linked subunits F1-F2, which is essential for the progeny virus to become infective (32). Previous studies have demonstrated that the amino acid composition of the F protein cleavage site is a key determinant of NDV tissue tropism and virulence. Virulent strains of NDV contain multiple amino acid F protein cleavage sites, which are cleaved by proteases widely present in host cells and tissues, which ultimately has consequences like tissue necrosis and virus spread (33, 34). In contrast, the F protein cleavage site of low virulence or avirulent pathotypes, NDV strains are usually monobasic or dibasic amino acid residues, which are cleaved by trypsin-like enzymes that only exist in the respiratory and intestinal tract. F protein of other tissues or cells is not sensitive to intracellular proteases and is not cleaved into F1 and F2. (35).

The HN protein is located on the surface of the virus envelope and is abundant in content, which affects the pathogenicity, replication, and biological characteristics of the virus. HN is a multifunctional molecule that recognizes cellular sialic-acid-containing receptors and has neuraminidase (NA) activity to hydrolyze the sialic acid molecules from progeny virus particles, prevent viral self-aggregation, and interact with F to promote fusion and other functions (36, 37). The HN is present as a homotetramer with disulfide linked dimers within virus infected cells, which include a stalk region and a large globular head domain (38). The globular head region of the HN protein is considered the antibody binding site, and the main amino acid residues that bind to sialic-acid-receptors and NA activities are located in the globular head (39, 40). Deng et al. suggested that the stalk domain of HN protein confers specificity for fusion, mediates the interaction of HN with the homologous F protein, and further fusion with the virus-specific membrane, and affects its fusogenic activity (41).

The L protein, which contains all the catalytic activities associated with viral polymerases, is the largest protein in the NDV genome and the last gene to be transcribed during the viral replication cycle. The existence of two forms of L protein of NDV isolates due to a compensatory mutation in mutation domain V. The L polymerase protein of non-segmented, negative-stranded RNA viruses is a multifunctional protein that has all the activities required to catalyze genome replication and post-transcriptional modification of mRNA (42, 43). Two methyltransferase motifs (G-G-D and K-D-K-E motifs) were found in the CR region of the L protein of the NDV virus. They catalyze the methylation of the G-N-7 position of the mRNA cap structure, which is necessary for the efficient translation of mRNA (42, 44). L protein modulates the virulence of NDV, and its role in the virulence of the virus may be achieved by increasing the rate of viral RNA synthesis during replication.

### Infection mechanism

**Figure 3:**
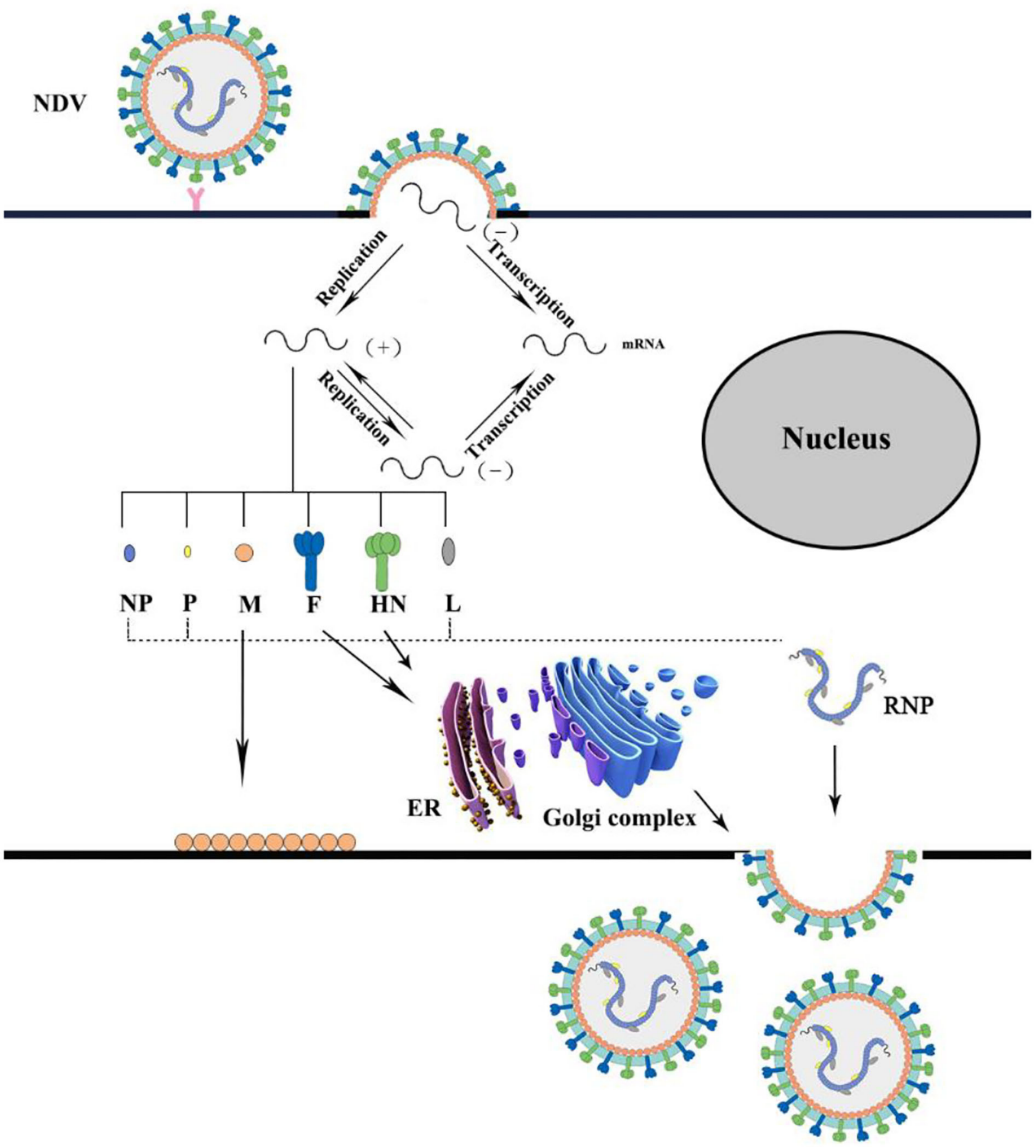
Schematic representation of Newcastle disease virus replication.

The entire process of NDV infecting host cells from completing its replication and producing infectious progeny viruses relies on major structural proteins to complete. The infectivity of NDV depends on the cleavage of the F protein precursor F0 to the F1–F2 subunits. Therefore, the amino acid sequence of the F protein cleavage site is considered to be the main determinant of infection. HN protein is related to virus attachment and into the host cell. The attachment stage of NDV-infected cells is through the combination of HN glycoprotein on the surface of the virus and the sialic acid-containing receptors on the host cell surface to infect respiratory epithelial cells, which is the first step of NDV infection (45). Then, the F protein of the viral envelope is responsible for the fusion of the viral envelope and the cellular plasma membrane to allow the entry of the nucleocapsid into the cell cytoplasm. In addition, infections also occur through receptor-mediated endocytosis and sometimes through caveolae-dependent endocytosis. Simultaneously, the viral RNA is encapsidated by NP protein to form an N-RNA template, which is mediated by the P protein and catalyzed by the L protein to form an active RNP complex to enter the cytoplasm. Following the entry into the host cell cytoplasm, the M protein is separated from the polymerase complex to transcribe the viral genome. The negative-sense RNA genome is transcribed into positive-sense mRNA, which is then translated into viral proteins (46). The M protein is required for the assembly of virus particles and the budding process of progeny (47, 48). Finally, the HN protein promotes the detachment of the virus from the cell and removes sialic acid molecules from progeny virions to prevent self-aggregation during budding (34) (Figure 3).

The attachment stage of NDV-infected cells is through the combination of HN glycoprotein on the surface of the virus and the sialic acid-containing receptors on the host cell surface to infect respiratory epithelial cells. Then, the F protein on the viral envelope is responsible for the fusion of the viral envelope and cellular plasma membrane to allow the entry of the nucleocapsid into the cell cytoplasm. The negative-sense RNA genome is transcribed into positive-sense mRNA, which is then translated into viral proteins. Finally, the HN protein promotes the detachment of the virus from the cell and removes sialic acid molecules from progeny virions to prevent self-aggregation during budding.

## Viral isolation

Viral isolation has been the standard strategy compared with all other strategies, which is important for the prediction and control of epidemics, as well as for vaccines and antiviral drug development. The viral isolation strategy has been widely and nearly universally performed since the early 1960s and has always been considered the “gold standard” for the detection of viruses (49). The isolation of NDV is capable of being undertaken through the inoculation into specific pathogen-free (SPF) chick embryo, chicken embryo fibroblasts (CEFs), chicken embryo kidney cells (CEKs), Hela cell lines, and Vero cell lines, among which CEFs are used more frequently. The respiratory secretions in the initial stage of infection were collected aseptically the case before death, and the spleen, brain, and lung tissues were collected aseptically after the culling of animals to prepare sterile inoculation materials. The diseased material is prepared into an emulsion with antibiotic-added physiological saline or phosphate buffered solution (PBS) and the precipitate is centrifuged to obtain the inoculation material. At the same time, take a small amount of inoculum in a tube of broth, blood agar slant, and anaerobic liver soup for sterility inspection, and culture and observe at 37°C for 2–6 days. If there is not any growth of bacteria, inoculate chicken embryos or cells. Method 1: the allantoic cavity of 9~11 day-old SPF chicken embryos was inoculated with sterile materials and incubated for 5 days for virus culture. The allantoic fluid of chicken embryos was collected aseptically from embryos that died more than 24 h post-inoculation for virus identification. Method 2: CEFs (or Hela cells, Vero cells) were inoculated with sterile materials and cultured in a 36°C incubator. If cytopathic effects (CPE), mainly in the form of rounded, fused. and granulated giant cells rapidly detaching from the monolayer, are observed by the electron microscope within 24 to 48 h of inoculation, then collect the cell sap for virus identification. In practice, however, virus isolation is laborious and time-consuming, making it less suitable for testing large numbers of samples that are obtained in clinical trials.

## Serological assays

The body generates immune responses to produce corresponding specific antibodies for defense when NDV infects the body. B cells (bone marrow, spleen, and lymph nodes) in the lymphoid tissues of the body are stimulated by antigenic substances to proliferate and transform into plasma cells, which in turn produce immunoglobulins that bind to cognate antigen (50). Serological assays based on the interaction between antigens and antibodies are an important strategy for the detection of NDV and are typically used as the first line of testing. Therefore, there is an urgent need for highly specific and sensitive serological testing methods for the diagnosis of NDV in clinical settings in endemic areas. Currently, a large number of serological assays have been developed for the detection of NDV, and some commonly used strategies are presented, such as hemagglutination and hemagglutination-inhibition tests (HA-HI), enzyme linked immunosorbent assay (ELISA), neutralizing antibodies test (NT), Immunofluorescence assay (IFA) and Immune colloidal gold technique (GICT).

### Hemagglutination and hemagglutination-inhibition tests

**Figure 4:**
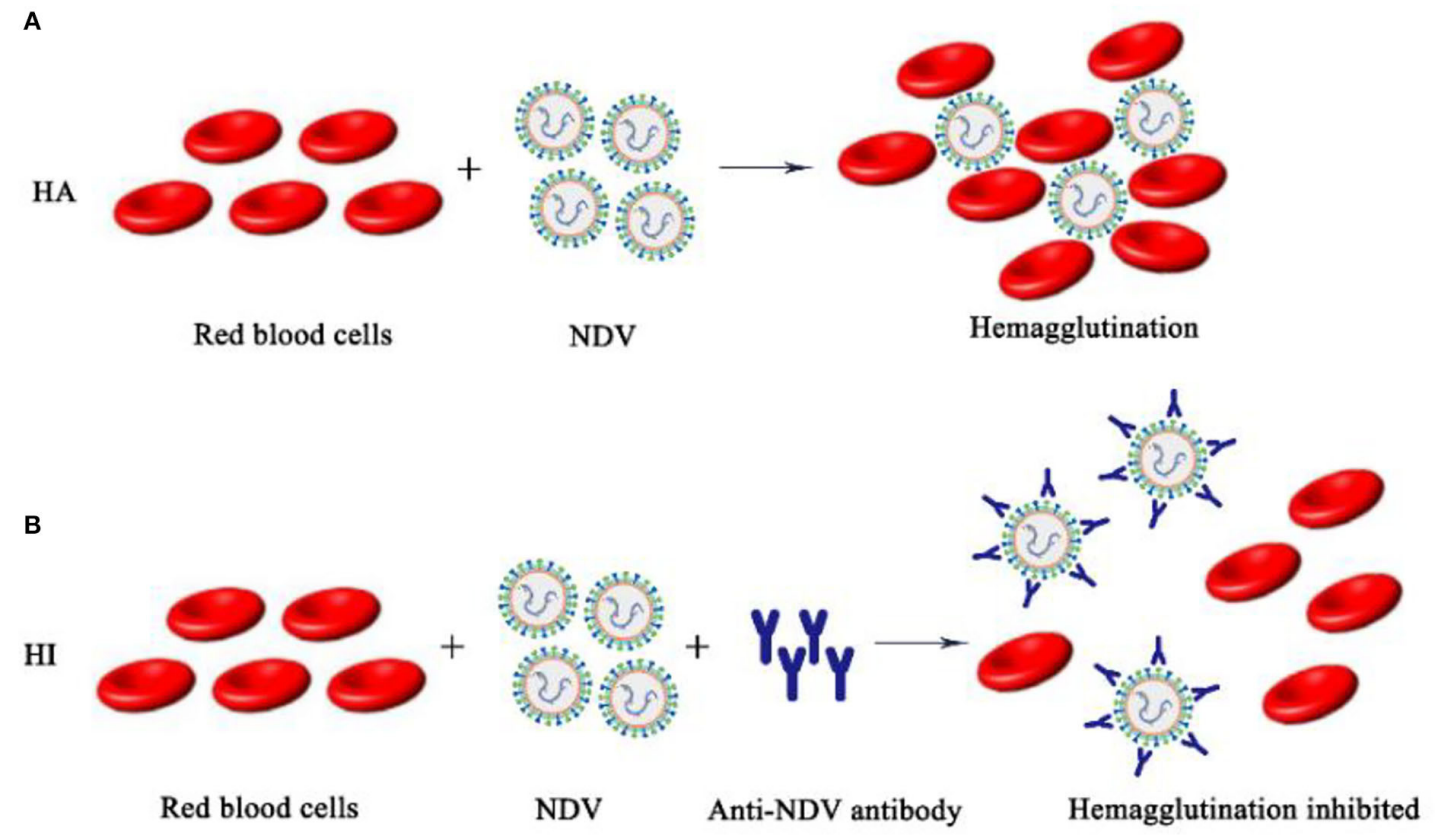
Schematic diagram of NDV HA-HI. **(A)** NDV hemagglutination mechanism; **(B)** NDV hemagglutination inhibition mechanism.

The surface of the NDV envelope exhibited both hemagglutinin and neuraminidase activities. Hemagglutinin will agglutinate different animals' red blood cells *in vitro* (mainly chicken red blood cells), and neuraminidase dissociates agglutinated red blood cells to release NDV. According to the biological characteristics of NDV, the isolated NDV was identified by HA-HI tests, and the hemagglutination titer and maternal antibody titer were determined (51). However, there are other pathogens such as AIV that have hemagglutinating chicken red blood cell function, so the HA test alone cannot make a definite diagnosis. The role of NDV in agglutinating red blood cells is blocked by binding to specific antibodies. Therefore, it is necessary to use a known antiserum for the HI test to make a clear identification of the newly isolated virus. The HA-HI method may be used to identify unknown viruses utilizing antibodies of known specificity, and may also be used to identify corresponding antibodies in serum utilizing viruses of knew (Figure 4).

Judgment of HA test results: The isolated NDV to be tested was diluted in a gradient and then added to chicken erythrocyte suspension, and a PBS negative control group was set at the same time. The result is judged when the PBS single well settles to form a button. Tilt the reaction plate and observe whether the RBC are completely agglutinated. The maximum dilution of allantoic fluid that resulted in complete agglutination of RBC suspension was recorded as HA unit (HAU) of virus titer. HI test was performed with standard positive serum for hemagglutination test positive samples. Judgment of HI test results: The NDV antigen to be tested was added to the DNV-positive serum diluted in series and mixed, and then the RBC suspension was added. The result is judged when the PBS single well settles to form a button. Tilt the reaction plate, if the RBC are observed to flow down like teardrops, it indicates that the sample to be tested is NDV.

### Enzyme linked immunosorbent assay

**Figure 5:**
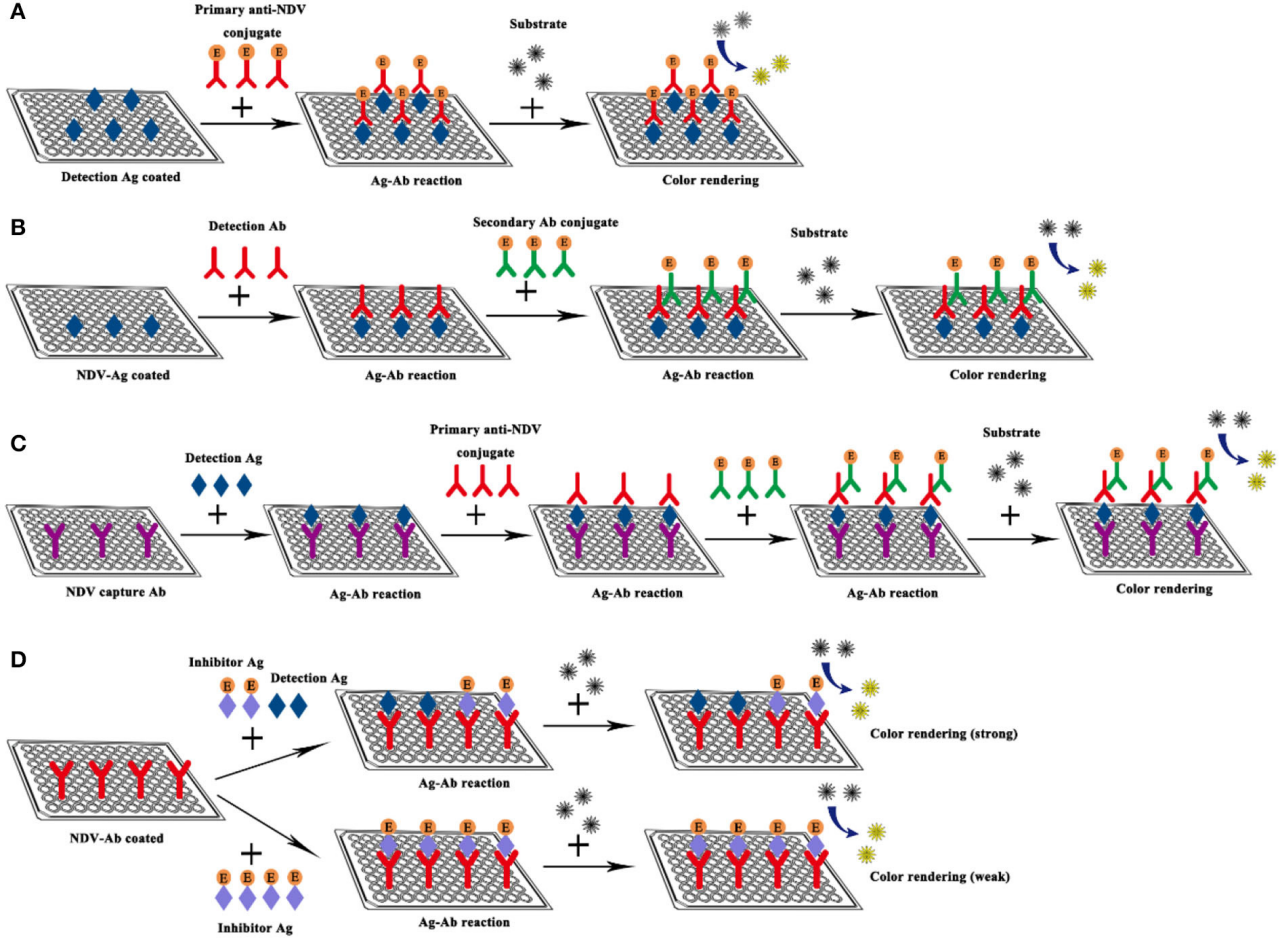
Schematic diagram of NDV ELISA. **(A)** Direct ELISA; **(B)** Indirect ELISA; **(C)** Sandwich ELISA; **(D)** Competitive ELISA.

ELISA refers to a qualitative and quantitative detection method that binds soluble antigens or antibodies with solid-phase carriers such as polystyrene and uses the specific recognition and binding effect of antigen and antibody to carry out immune reactions. The ELISA technique has high specificity, sensitivity, and the ability to directly detect complex biological samples, it is regarded as an effective strategy for detecting specific antibodies in serum, and has been widely used in the detection of NDV (52–56). Here, taking the indirect ELISA detection method as an example, the samples to be tested are added to a solid-phase carrier that has attached the antigen. If the serum sample to be tested contained antibodies against NDV, these antibodies will be absorbed from the serum sample, which will specifically bind to the antigen on the solid carrier to form an antigen-antibody complex (57). Then the secondary antibody (the antibody tagged with the enzyme like horseradish peroxidase) and substrates are added to produce color. The color of products is directly related to the amount of the corresponding antibody in the sample, which can be analyzed qualitatively or quantitatively according to the depth of color reaction (Figure 5).

Traditional ELISAs that use polyclonal and monoclonal antibodies as reagents have the disadvantages of limited quantities, difficulty in permanent storage, and the need to use secondary antibodies (58). Nanobodies are fused with multiple tags in their tertiary structure by recombinant technology and expressed with different expression systems, thereby providing an effective detection method for diagnostic purposes. The ferritin-fused nanobody (fenobody) and nanobody-fused reporter (RANbody) have been designed and derived from the nanobody for developing the diagnostic immunoassays. Ji Pinpin et al. established a sandwich ELISA detection method using fenobody as the capture antibody and RANbody as the detection antibody. This method uses NDV as the antigen to screen 13 nanobodies that bind specifically to NDV particles from immunized Bactrian camels. Five fenobodies and RANbodies derived from nanobodies were selected and the optimal pairing concentrations of fenobodies and RANbodies were determined. This sandwich ELISA was utilized to detect 150 clinical samples, which were collected from tracheal, cloacal swabs, allantoic fluid, chicken sera, cell culture medium, and tissue samples to determine their cut-off values. The results show that an OD450mm of 0.0895 is considered the cut-off value of ELISA, samples with OD450 higher than 0.0895 are considered positive, while conversely, the samples were negative. The OD450nm value was above 0.0895 when the HA titer of NDV in the allantoic fluid was 22 or the amount of purified NDV particles was 10 ng. It exhibits 98.7% agreement with the HA test. The specificity results showed that the OD450nm values of other poultry viruses, H9N2, H5N1, H7N9, IBV, IBDV, FADV, and ALV-J were all less than 0.058. In addition, this method can detect different class II strains (LaSota, F48E9, and sx10), but cannot detect class I strains (QH-1), which have good specificity (59).

To detect NDV antibodies in chicken serum more accurately and specifically, Sun established a competitive ELISA (cELISA) detection method using nanobody-horseradish peroxidase (HRP) fusion protein as a probe. First, NDV-NP proteins were induced to express and purify, and then 9 anti-NDV-NP protein nanoantibodies were screened from immune Bactrian camels. Screen the best nanobody to develop cELISA. In the analysis of clinical serum samples by cELISA, the sensitivity and specificity of this method are 100 and 98.6%. After the 368 clinical chicken sera and 108 egg yolks were tested for anti-NDV antibodies separately with the HI test, commercial ELISA kit, and cELISA. The consistency of the HI test, commercial ELISA kit, and cELISA was 97.83 and 98.1% when testing clinical chicken sera and both agreed to 100% when testing egg yolks, which were highly consistent. However, cELISA showed higher sensitivity when detecting anti-NDV antibodies in the sequential sera from challenged chickens. In addition, a close correlation was found between the percent competitive inhibition values of cELISA and HI titers (R2 = 0.914) (60).

A Ahmed has established V-ELISA and P-ELISA to detect antibody against the C-terminal region of the NDV non-structural protein V and structural protein P (61). D.D. Oliveira has developed an indirect ELISA for the detection of NDV specific Japanese quail IgG (62). Besides, Xue Songguo and Zhang Anding respectively established ELISA methods to detect NDV. The ELISA test method is simple to operate and the test results are obtained in a short time. However, commercial ELISA kits with stable detection effects are more expensive, leading to higher detection costs. Therefore, the development of new ELISA kits is needed to reduce costs.

### Neutralization test

HI, and ELISA assays are used to measure protection and immune response against NDV but do not corroborate the presence of neutralizing antibodies (nAb). The neutralization test (NT) refers to the loss of pathogenicity to susceptible animals after the virus or toxin is combined with the corresponding antibody. NT mainly has simple qualitative tests, fixed serum dilution virus method, fixed virus dilution serum method, and virus plaque reduction method. The NT method may also be used to identify unknown viruses utilizing anti-NDV serum of known specificity; however, most often, this test is applied to detect whether the serum contains specific antibodies.

**Figure 6:**
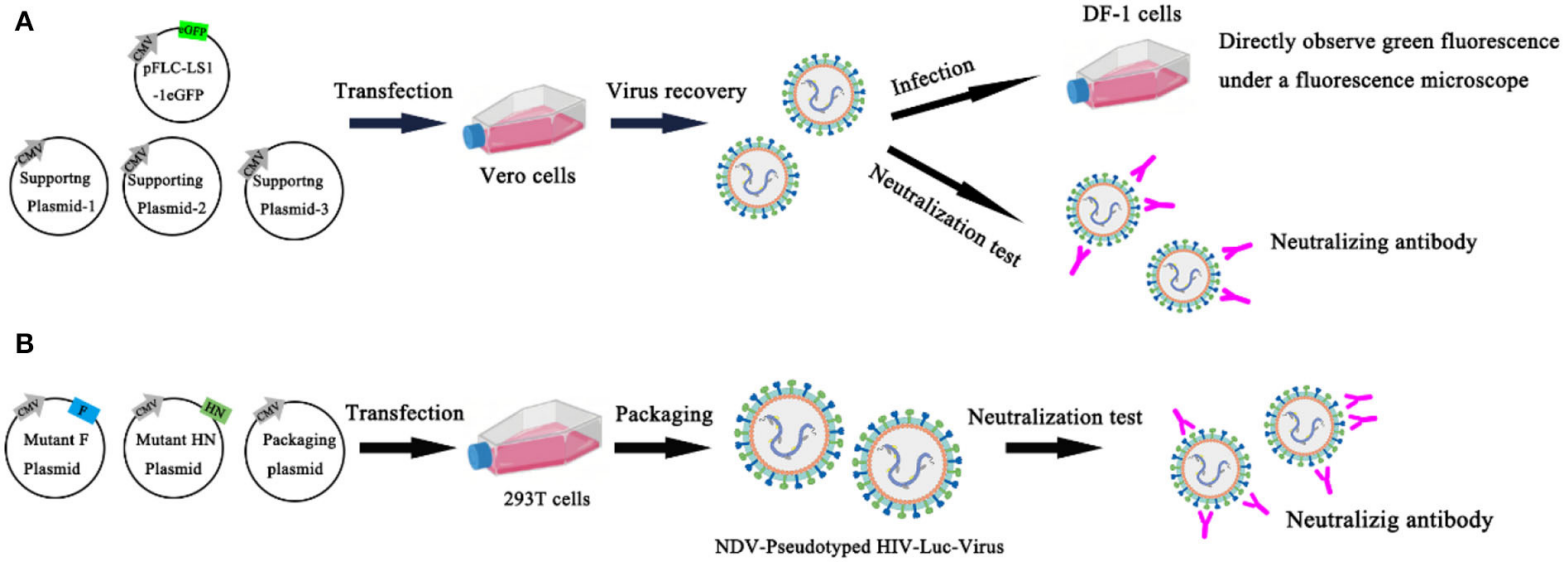
Neutralization test. **(A)** An eGFP-based neutralization test (eGFP-NT) was developed by Chumbe Ana; **(B)** Bin Wang established a novel neutralization assay to evaluate neutralizing antibodies against NDV with the NDV-pseudotyped HIV-Luc viruses.

Chumbe et al. (63) developed an eGFP-based neutralization test (eGFP-NT), in which nAbs titers were expressed as the reciprocal of the highest dilution that expressed the eGFP. In this method, the GFP reporter gene was inserted basis on conventional NT, and the rLS1-1eGFP reporter NDV was established and identified. Compared with conventional NT, eGFP-NT shortens the turnaround time and gives conclusive results within 24 h without using any additional staining procedures. This method evaluates the effect of the rLS1-1eGFP virus in measuring nAb titer by testing 57 serum samples, and the results are significantly correlated with conventional NT. All serum samples were heat inactivated (56 °C for 30 min) and stored at −20 °C. 57 chicken sera were tested for anti-NDV antibodies separately with the HI method, commercially ELISA kits, conventional NT, and eGFP-NT to detect anti-NDV antibodies. The results showed that the conventional NT had a significant correlation with HI (R = 0.816) and ELISA (R = 0.791). However, eGFP-NT shows a higher correlation (R = 0.994) and is a more accurate method to quantify nAbs (63) (Figure 6A). Bin Wang et al. established a novel neutralization assay to evaluate neutralizing antibodies against NDV with the NDV-pseudotyped HIV-Luc viruses. In this new Luc report neutralization test, the measurement of luciferase activity after pseudovirus infection and the TCID50 assay is usually used to titrate the NDV pseudotyped HIV-Luc virus. First, construct and express wild-type and HN/F mutant of NDV in 293T cells to produce NDV-pseudotyped HIV-Luc viral particles. It is found that the infection efficiency of the NDV-pseudotyped HIV-Luc virus was over 34-fold lower than with the VSV-G-pseudotyped HIV-Luc virus. The cytoplasmic domain of HN may be the main reason for the lower infection efficiency. To determine whether NDV pseudotyped HIV-Luc virus can be used to measure neutralizing antibodies against NDV. In this experiment, three NDV immune sera were 2-fold serially diluted and incubated with an equal volume of DNV-pseudotyped HIV-Luc virus (100 TCID50) for 1 h at room temperature. The analysis of luciferase showed that the NDV-pseudotyped HIV-Luc virus system was successfully used to detect NDV neutralizing antibodies. The NT titers of 16 NDV immune sera against NDV were successfully detected by both neutralization assays by both the NDV-pseudotype HIV-Luc neutralization test and the conventional VN test. Moreover, the NT titer determined by this method was 2–5 times higher than that determined by the conventional VN test (64) (Figure 6B). The advantages of the NT are high sensitivity and specificity, the existence of neutralizing antibodies in the body for a long time, and the neutralizing antibodies of most viruses are directly related to immunity. The disadvantage of the NT is that it uses a live host system, and it takes a certain amount of time for the virus to act on the host system, so the results are slow.

### Immunofluorescence assay

The IFA as a confirmatory tool is another indicator for the detection of NDV, due to the uncertainty of ELISA results (Including false negatives and false positive results). The IFA method uses a fluorescein-labeled secondary antibody such as isothiocyanate (FITC) as a fluorescent probe and then observes the fluorescence with a fluorescent microscope to analyze the corresponding antigen or antibody. As early as 1961, IFA has been used for the rapid diagnosis of human influenza. Compared with other enzyme labeling techniques, the inherent antigen of IFA is better preserved, and it is better to avoid antigen contamination in the blood and avoid the interference of endogenous antibiotics. In addition, it has the advantages of strong specificity, high sensitivity, and fast speed. However, the problem of IFA non-specific staining has not been completely solved, the objectivity of the results is insufficient, and the technical procedures are still relatively complicated.

**Figure 7:**
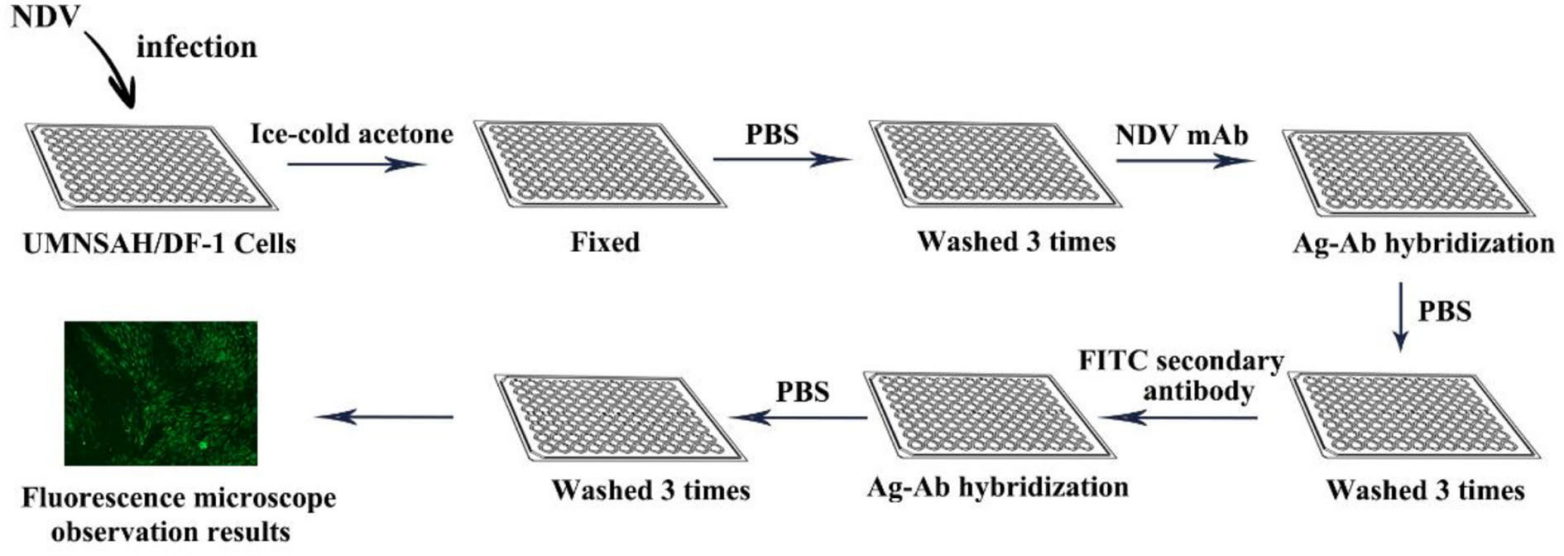
Schematic illustration of indirect immunofluorescence of NDV.

Skeleles used IFA for AIV testing for the first time when avian influenza broke out in Asia in Pennsylvania in 1984. Initially used for virus identification and localization of specific antigens in virus-infected cells, mainly intranuclear fluorescence. Studies have shown that CEFs, CEKs, Hela, Vero, and human amniotic epithelial cells (hAEC) are usually infected with NDV for IFA detection. The first step of IFA is that the UMNSAH/DF-1 cells are infected with NDV and fixed by ice-cold acetone. Then, the infected cells are incubated in the diluted mAb (set in a negative control group at the same time), and the cells were washed 3 times with phosphate-buffered saline (PBS) after incubation for half an hour at 37°C. Subsequently, cells were treated at 37°C using the diluted FITC-secondary antibody for 30 min. Finally, after washing with PBS five times, the positive cells were detected by a fluorescence microscope (Figure 7).

Sahib et al. (65) used immunofluorescence to determine the effect of intratumoral co-administration of oncolytic NDV and bacterial hyaluronidase in tumor models. The immunofluorescence results of the combined treatment of virus and bacterial hyaluronidase show reduced growth of primary tumors treated without increasing the metastatic potential and in prolonged survival of the tumor-bearing animals (65). The HN was surfaced displayed on Lactococcus lactis by Baradaran et al. (66) and the immunofluorescence assay showed the binding of the recombinant HN-AcmA protein on the surface of the bacterial cells. Further study of its cancer targeting ability through the connection with MDA-MB231 breast cancer (66).

### Immune colloidal gold technique

**Figure 8:**
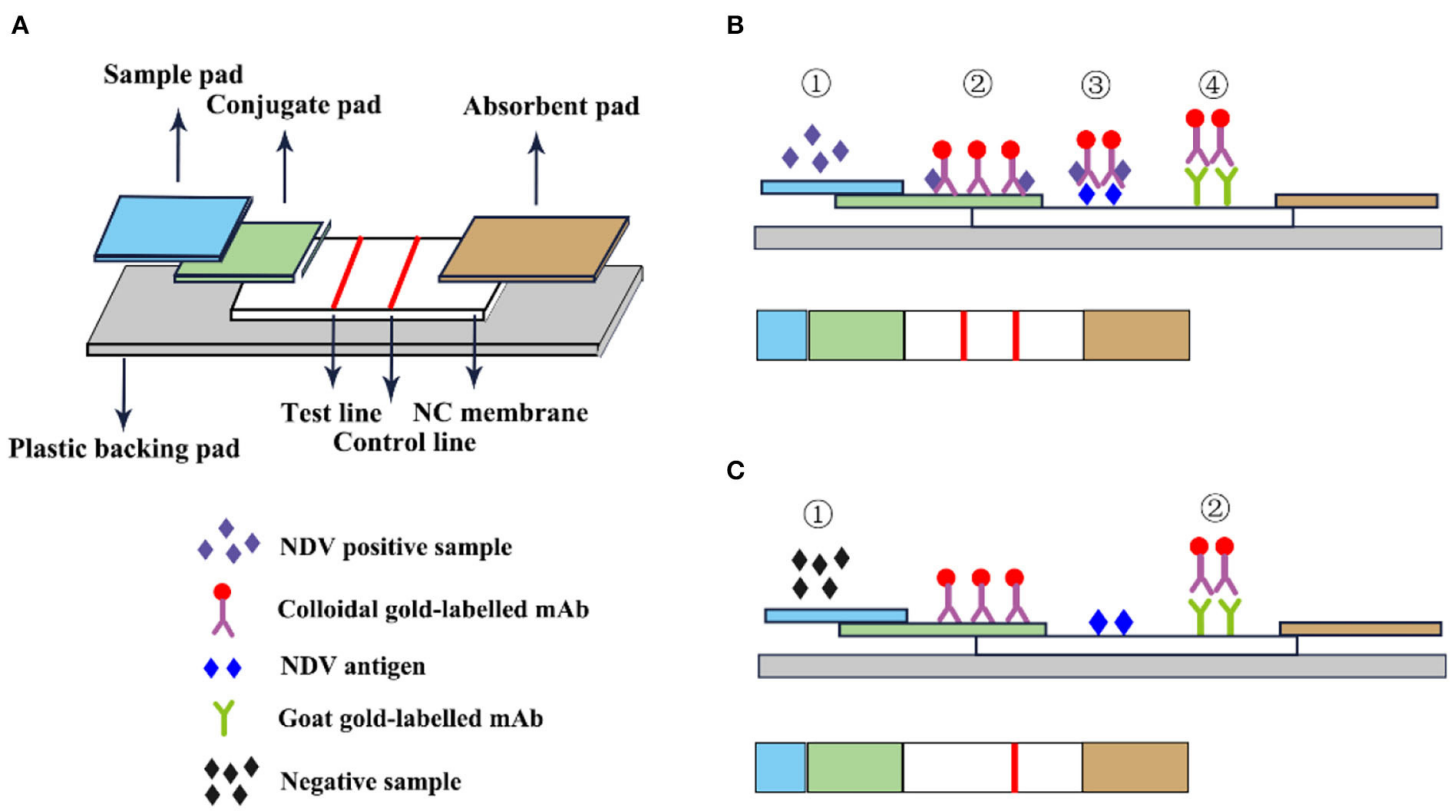
Schematic diagram of immunocolloidal gold. **(A)** Immunocolloidal gold components; **(B)** NDV positive control; **(C)** Negative control.

The immune colloidal gold technique is a new type of immuno-labeling technology developed in the 1980s following the three major labeling technologies of immunofluorescence technic, radiation isotopic tracer technique, and enzyme labeling (67). Among them, the most widely used is colloidal gold immunochromatography. GICT has become one of the most widely cited immunological detection techniques today, due to its single detection, special sensitivity, no need for auxiliary reagents and instruments, visual observation of the results, and long-term storage of the results. It is especially suitable for basic units, field operations, time-critical inspections, and large-area general surveys. The immunochromatographic test strips are assembled from top to bottom with a colloidal sample pad, gold-label pad, nitrocellulose membrane, and absorbent pad (68). The gold-labeled pad is to fix the colloidal gold-labeled antibody or antibody on the glass cellulose membrane through a desiccant, and specifically bind to the detection species antigen or antibody. The nitrocellulose membrane is a reaction carrier pre-coated with the detection and the control line and is used to combine with the colloidal gold marker to produce an immune reaction resulting in the color development of the corresponding detection line (Figure 8).

Li et al. (69) used HN mAb labeled with colloidal gold as the detector and developed an immunochromatographic strip for the detection of NDV. In this experiment, HN mAb labeled with colloidal gold was distributed to fiberglass pads to generate conjugate pads. Then the chicken anti-NDV pAb and staphylococcal protein A solution were distributed on the nitrocellulose membrane as the test line and the control line. The sensitivity test results showed that the immunochromatographic strip detected 10^4.9^ EID_50_ viruses/0.1 mL, and the classic HA test detected 10^5.2^ EID_50_/0.1 mL, which was equivalent insensitivity. The strip specifically recognized the NDV antigen and has no cross-immune reaction with the Infectious bursal disease virus (IBDV) vaccine (strain B87), Marek's disease virus (MDV) vaccine (strain Fc-126), Laryngotracheitis virus (ILTV) vaccine (strain K317), Infectious bronchitis virus (IBV) vaccine (strain W93), Egg-drop syndrome virus (EDSV-76; strain A V-127) and avian influenza A virus (AIV). In addition, this method also specifically recognizes the virulent and attenuated strains of NDV (69). F. Yang et al. developed an innovative gold immunochromatographic assay with a quantitative immunosensor, which has a two-reaction system and double-test lines. In this experiment, a standard curve was established to quantify DNV based on the optical density of the reference sample to quantify, and the antibody concentration was linear within the titer range of 22–29. The test strips and the HI method were used to detect NDV in 506 serum samples collected from chickens. The results of the test strips of 468 test samples were consistent with the HI method and the coincidence rate was 92.49%. The immune colloidal gold test strip has no cross-immune reaction with AIV, EDS, IB, and IBD which indicated that the test strips had high specificity and could specifically detect NDV antibodies. This method overcomes the limitation that the existing colloidal gold immunochromatographic method is only used for qualitative or semi-quantitative purposes (70). GICT technology is easy to operate and does not require other instruments. Results are available in minutes and can be tested anytime, anywhere. It has become one of the most widely used detection technologies.

## Molecular assays

The cross-reaction between NDV and some avian paramyxoviruses leads to uncertainty in ND serological testing. Traditional serological testing has an insurmountable barrier in typing. Therefore, people hope to establish a standard at the molecular level to analyze the differences between various NDV strains. Molecular assays have played an important role in quickly identifying NDV and distinguishing it from other closely related pathogens. In this review, we introduced reverse transcription polymerase chain reaction (RT-PCR), real-time quantitative RT-PCR (qRT-PCR), and loop-mediated isothermal amplification (LAMP) diagnostic methods.

### Reverse transcription polymerase chain reaction

The RT-PCR with high sensitivity and high selectivity is consideredthe gold standard for the molecular amplification and assay of the virus. RT-PCR technology is a method to selectively amplify RNA fragments *in vitro*. First, reverse transcription of RNA virus nucleic acid to synthesize cDNA, and then perform PCR amplification. RT-PCR has the advantages of specificity, rapidity, sensitivity, and simplicity.

Jestin et al. (71) successfully established an RT-PCR method to identify NDV for the first time. The sequence selected for amplification consists of 238 bp lying in the gene encoding the fusion protein F. The results showed that 27/30 A-PMV1 strains obtained a positive signal of 275 bp. The other three strains showed fragments that were smaller than expected, at about 230–250 bp, probably due to the loss of the NarI site. At the same time, the other avian paramyxoviruses tested gave no signal (71). Li et al. (72) designed degenerate primers for the RT-PCR amplification of the main functional regions of the NDV fusion protein gene in air sample swabs based on published primers. Air samples were collected by liquid impingement *via* AGI-30 samplers operating in the center and four corners of each house. Three consecutive samples were taken at the same sampling point in each house. Avirulent viruses were detected both in air samples and swab samples from four houses and virulent viruses were detected only in air samples from two rooms by RT-PCR based on degenerate primers. This technique could reduce the time required to identify NDV infected flocks while distinguishing between virulent and avirulent viruses (72). Desingu PA has developed a novel HAd-based sensitive RT-PCR technology utilizing the hemosorption characteristics of NDV. Chicken red blood cells (RBC) are added to the supernatant of clinical samples such as allantoic fluid, tissue tritium, cloaca, and tracheal swabs infected with NDV to adsorb the virus on the surface of RBC. The red blood cells that adsorb the virus are detected by RT-PCR. This method shows 100 times higher sensitivity than the traditional RNA extraction and RT-PCR detection systems and can be exploited in the case of clinical samples with low NDV load (73). Yi et al. (74) designed a pair of primers that was highly homologous to three NDV pathotypes based on the shared nucleocapsid protein (NP) gene sequence and established a red blood cell adsorption-coupled RT-PCR assay. This method was used to detect stool, liver, kidney, and lung samples, and the results showed that the limit of NDV detection was in the range of 10^7^ fold diluted samples (100 EID_50_). This method has higher sensitivity and is suitable for the detection of NDV in different types of samples (74).

### Real-time qRT-PCR

**Figure 9:**
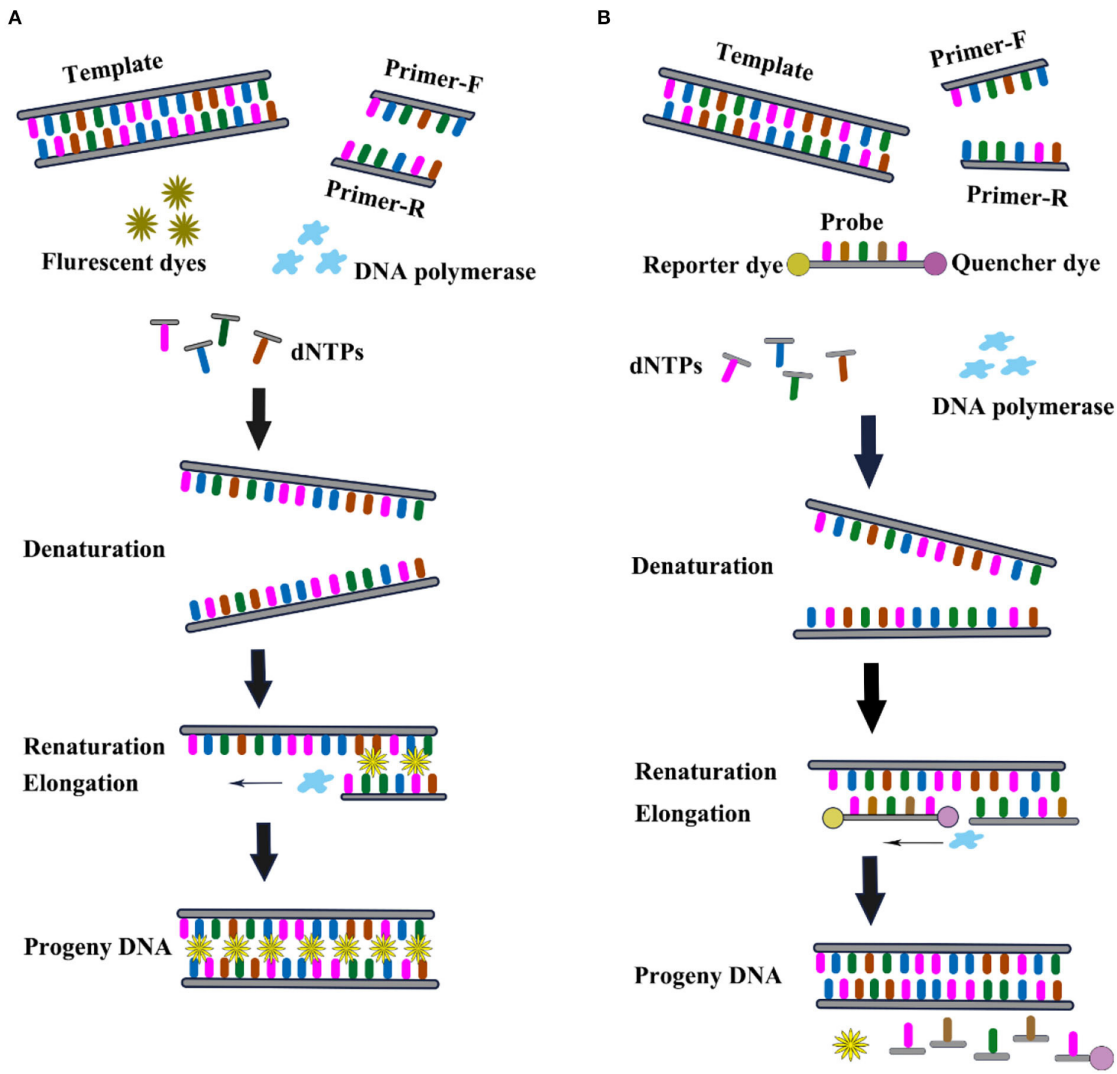
Schematic diagram of real time qRT-PCR. **(A)** NDV SYBR Green I assay. **(B)** NDV TaqMan assay.

The invention of real-time qRT-PCR technology represented another revolutionary leap forward in the field of DNA analysis. The key feature of the real-time qRT-PCR technique is the ability to monitor when the DNA amplification process occurs, and finally, use the standard curve to quantitatively analyze the unknown template. Compared with conventional RT-PCR, real-time qRT-PCR has several advantages such as rapidity, high sensitivity, low false positives, and the possibility of quantitative analysis. In real-time qRT-PCR, the DNV RNA needs to be extracted from infected samples and then added to the reaction mixture for amplification. The reaction mixture includes necessary factors such as fluorescent dyes, reverse transcriptase, DNA polymerase, dNTPs, and specific primers. As the target gene is amplified, the fluorescence in the sample increases as the number of products increases. The Ct value is defined as the PCR cycle number and is inversely proportional to the starting amount of target cDNA. The real time qRT-PCR can be divided into TaqMan and SYBR Green I based on different chemistry analysis. SYBR Green I is a dye that binds to all double-stranded DNA molecules regardless of sequence. The fluorescence increases significantly when the SYBR Green I dye intercalates into dsDNA. It has the advantages of low cost and simple experimental reagents. The TaqMan (small groove binder) probe provides a turnkey homogeneous chemical method for the detection of single nucleotide polymorphisms (SNP) with high specificity. Real-time qRT-PCR is a powerful tool for the detection and quantification of gene expression owing to its high sensitivity, specificity, reproducibility, and quantitative analysis capabilities (Figure 9).

To diagnose ND simply and quickly, Wenjing Wang has established two detection methods based on reverse transcription recombinase-aided amplification (RT-RAA) technology. One of the detection methods is the real-time fluorescence-based reverse transcription which uses RT-RAA and exo probes for recombinase-aided amplification. The reaction temperature and time of the method were optimized, and its specificity and sensitivity were tested. This method had no cross reactivity to the nucleic acids of other avian pathogens and does not require RNA reverse transcription. The experiment is completed by one-step method, which could complete the reaction within 26 min, and is 100 times more sensitive than conventional PCR methods (75). The developed triplex real-time qPCR by Zhang et al. (76)is fast, specific, and sensitive, and is feasible and effective for the simultaneous detection of AIV, NDV, and DTMUV in ducks. The results showed that the detection limit of the triple real-time PCR was 1 × 10^1^ copies/μL, which was at least 10 times more sensitive than conventional PCR methods. In addition, the triple assay will not cross-react with other duck pathogens and is highly specific. Taking virus isolation as the gold standard, the diagnostic positive predictive value and specificity of the three viruses were all above 85%, while the diagnostic sensitivity and negative predictive value were both 100% (76). Liu et al. (77) established a qRT-PCR method for the rapid, on-site detection of NDV on the farm. In the field conditions, NDV nucleic acid was manually extracted with minimal equipment and used the portable T-COR4 platform for q-RTPCR detection. Forty-two RNA samples of NDV were collected from the farm, 15 positive samples were detected by real-time qRT-PCR on the portable T-COR platform, and reached an agreement of 83% when the same RNA preparations were tested in the Rotor Gene thermocycler under the laboratory setting (77). Wise et al. (78) designed three sets of primers and probes for the F gene and M gene fragments of NDV, and a qRT-PCR was developed to detect NDV. A first primer-probe set was designed to detect sequences from a conserved region of the M gene that recognized a diverse set of NDV isolates, enabling the detection of 10^3^ genome copies. A second set was targeted to sequence in the F gene that codes for the cleavage site and distinguish potentially virulent NDV isolates, enabling detection of 10^4^ genome copies. The third set of primer probes is aimed at the M gene and is used to detect the genotype of North America before 1960, which includes common vaccine strains in the United States, enabling the detection of 10^2^ genome copies. Both M gene assays detected approximately 10^1^ 50% of the egg infectious dose (EID_50_), while the F gene assay detected approximately 10^3^ EID_50_. At the same time, the virus isolation and identification method were used for detection, and the results showed that a positive correlation was obtained between qRT-PCR and virus isolation for NDV from clinical samples (78). Zeynalova et al. (79) monitors Avian Influenza (AI) and NDV in the Barda Region of Azerbaijan using real-time qRT-PCR and hemagglutination inhibition. A total of 1,030 swabs and 3,890 blood samples were collected for the test. The qRT-PCR results showed that all swab samples were negative for AI and NDV. All blood samples were negative for H5 by HI, while 6.2% of all samples were positive for exposure to NDV (79).

### Loop-mediated isothermal amplification

LAMP performs nucleic acid amplification in a short time (usually within 1 h) under isothermal conditions, which is a simple, fast, accurate and low-cost gene amplification method. It is simpler and more convenient than PCR technology in terms of practical operation and instrument requirements. However, LAMP cannot be used for multiplex amplification, and is inferior to qPCR in sensitivity and quantitative ability.

A novel LAMP method for rapid detection of NDV by using one set of specific primers targeting the fusion protein genes was established by Hang Minh Pham. This method rapidly amplifies the target gene within 2 h, and only requires a conventional laboratory water bath or heat block for reaction. The 38 DNV strains, other different viruses, and clinical samples of experimentally infected chickens were used for genome detection to analyze the specificity and sensitivity of the LAMP method. The sensitivity test results showed that the detection limit of nested PCR or LAMP was 0.5 pg or 9 × 104 copies reaction determined by using a serial 10-fold dilution of the plasmids. The specificity results showed that all 38 strains of NDV gave a positive reaction, while no DNA band of three other serotypes of APMV and three other clinically related viruses (avian reovirus, fowl pox virus, and Marek's disease virus) was observed by both methods (80). Based on the previously reported LAMP method to detect NDV, Li et al. (81) have developed a One-step RT-LAMP assay for sensitive and specific detection of NDV. This method checks the DNA product from the RT-LAMP reaction by adding magnesium ions or SYBR Green I and realizes the visualization of the results. The improved RT-LAMP assay detected all 21 NDV isolates were positive and had no cross-reaction with bronchitis virus (IBV), laryngotracheitis virus (ILV), and low-pathogenicity avian influenza virus (LP-AIV). The sensitivity was 5-fold more sensitive than the previous LAPM and achieved 96.8% sensitivity with 62 samples. Therefore, the improved RT-LAMP assay is a rapid, simple, and cost-effective method that is practical for less well-equipped laboratories and in the field (81). By incorporating the optimized RT-LAMP and an LFA, a novel, user-friendly, simple multiple reverse transcription-LAMP-LFD strategy was developed by Xuan Wu's group for the highly sensitive detection tool which could simultaneously detect IBV and NDV visually. Compare the sensitivity and specificity of this method with conventional RT-PCR, nested RT-PCR (nRT-PCR), quantitative RT-PCR (qRT-PCR), and RT-LAMP monitored by electrophoresis. Based on the conserved regions of the IBV and NDV genomes, the IBV primers are located in the 5'-UTR region, and NDV primers are located in the conserved region of the LP gene. The advantage of mRT-LAMP-LFD analysis is that the test lines were observed when target templates were contained in the reaction mixture. However, conventional RT-PCR, nested RT-PCR, and qRT-PCR specific purpose bands are only when tested with target NDV templates. The sensitivity test showed that the lowest detection limits of mRT-LAMP-LFD were 100.8 IBV RNA copies/reaction and 100.7 NDV RNA copies/reaction. mRT-LAMP-LFD does not require special instrumentation, it is a promising qualitative detection tool and is suitable for some resource-poor areas (82). Recently, Liang et al. (4) developed a TaqMan loop-mediated isothermal amplification assay for the rapid detection of pigeon paramyxovirus type 1. This method targeted nine distinct regions of the F gene and designed six novel primers and a TaqMan probe. When the sensitivity of the TaqMan-LAMP assay was compared to that of conventional RT-PCR, it was found that the limit of detection was 10 copies μL-1 for PPMV-1 cDNA and 0.1ng for PPMV-1 RNA, both of which are more sensitive than conventional PCR. In addition, this detection method provides good specificity for NDV with no observed cross-reactions with other viruses. To evaluate the applicability of the assay, 108 clinical samples were used to compare the TaqMan-LAMP assay and commercial RT-PCR assay, and the concordance rate between the two methods was found to be 96.3% (4).

## Molecular assays

Proximity ligations assays, as a homogeneous immunohistochemical tool, enable the detection of protein modifications, endogenous proteins, and protein interactions with high specificity and sensitivity by using antibodies to detect two unique protein targets (83). A pair of PLA probes (antibodies equipped with DNA strand) binds the same target protein molecule and then the probes approach (84). Next, ligase and two additional oligonucleotides were introduced. These oligonucleotides hybridize with the PLA probes if in close enough proximity (~30–40 nm) to form a unique target DNA reporter molecule (85). The latter is a surrogate marker for the specific protein to be detected and contains a specific molecular barcode. Circularized padlock probes are amplified directly by either polymerase chain reaction (PCR) or rolling circle amplification (RCA) processes, taking advantage of SybrGreen real-time PCR amplification monitoring techniques for target detection (86). The PLA assays have been used to detect porcine parvovirus and foot-and-mouth disease virus (87). Boutheina Marnissi accurate detection of NDV using proximity-dependent DNA aptamer ligation assays. In order to analyze the performance of aptamer-assisted proximity connection, the reproducibility and the sensitivity of the homogenous and solid-phase PLAs tests were compared to those of sandwich ELAA and rRT-PCR. The results showed that solid-phase PLA with a LOD of 0.4 (EID_50_ mL^−1^) is three times more sensitive than the sandwich ELAA (1.2 EID_50_ mL^−1^) and one and a half times more than rRT-PCR (0.6 EID_50_ mL^−1^) and homogenous PLA (0.58 EID_50_ mL^−1^). Furthermore, the solid-phase PLA demonstrated a larger dynamic range with better values of LOD, the lower limit of quantification (LLOQ=0.1 EID_50_ mL^−1^), the upper limit of quantification (ULOD ULOD=10^5^ EID_50_ mL^−1^), and the minimal detectable dose (MDD=0.1 EID_50_ mL^−1^), as compared to the other assays. Sandwich ELAA, homogeneous, and solid-phase PLA were used to analyze 40 nasal/cloacal swabs collected from the farm, and the sensitivity and specificity of the developed method were compared with the gold standard rRT-PCR test. The results showed successful detection of 26 NDV positive samples out of 40 tested swabs, and the agreement with rRT PCR was 100%. Homogeneous and solid-phase PLA overcome limitations of laboriousness or lack of sensitivity of current methods for detecting viral genomes or proteins has a sensitivity similar to or higher than that of rRT-PCR. PLAs are high sensitivity, high efficiency, and reproducibility. They are reliable tools for detecting NDV and have the potential for a multiplex application for the detection of various avian viruses (88).

A sandwich electrochemical immunosensor was designed by Huang Jiaoling and established an electrochemical immunoassay for ultra-sensitive detection of NDV. The method is based on a gold nanoparticle-chitosan-graphene (AuNP-Chi-Gra) nanocomposite as the platform and adopts Cu (I)/Cu (II)-chitosan-graphene (Cu(I)/Cu (II)-Chi-Gra) nanocomposites as the label for detecting NDV. Graphene (Gra) has a large surface area and was used for the conjugation and the immobilization of anti-Newcastle disease monoclonal antibodies (MAb/NDV) and anti-Newcastle disease virus polyclonal antibodies (PAb/NDV), effectively increasing electrical signals. Cu (I)/Cu (II) was used as an electroactive probe, immobilized on a chitosan-graphite (Chi-Gra) hybrid material. It is detected by differential pulse voltammetry (DPV) after the sandwich-type immune response. A total of 120 clinical samples were assayed using the immunosensor and verified by the assays of virus isolation. The immunosensor results showed that 7 NDV positive samples were in agreement with the results of virus isolation and the detection limit line was 10^0.68^ EID_50_/0.1 mL. The novel immunosensor assay introduces Gra based on of PAb/NDV Cu(I)/Cu (II)-Chi (10^2.09^ EID_50_/0.1 mL), exhibited a linear response in a wider range (10^0.13^ to 10^5.13^ EID^50^/mL), had a lower detection limit (10^0.68^ EID_50_/0.1 mL). PAb/NDV-Cu(I)/Cu (II)-Chi-Gra is obtained by a facile fabrication procedure, has good selectivity, repeatability, stability, and is ultra-sensitive for the detection of NDV) (89).

## Conclusion and future prospects

While ND has been known since 1926, the disease is still endemic in many developing countries, spreading among different birds. The rapid spread of NDV may be caused by the respiratory tract, digestive tract, conjunctiva, injured skin, and cloacal mucosa. Caused by high fever, dyspnea, diarrhea, nervous disorder, mucosal and serosal bleeding as the main features. Therefore, there is an urgent need to develop rapid detection strategies and effective drugs for proper prevention and effective control of NDV. Furthermore, the molecular mechanisms of NDV-host pathogen interactions still require further studies to understand the NDV infection mechanism and to develop novel effective detection strategies and drugs to prevent and control NDV infections. At present, many serological and molecular biology strategies have been developed to detect NDV. However, both serological and molecular biological assays have some limitations. For the serological assays, the cross-reactivity between NDV and other homologous APMV viruses is a problem that may give a false positive. For molecular analysis, the missing unexpected sites may cause amplification failure. Thus, serological assays and molecular assays need to be further improved in terms of sensitivity and specificity to enhance detection stability and accuracy. In addition, most molecular and serological diagnostics are performed in a laboratory, requiring expensive equipment and reagents, specific maintenance requirements, and testing by trained personnel. Such equipment and expertise may not be available in all regions of the world, especially in economically underdeveloped regions. Thus, a simple, affordable, reliable, rapid, and diagnostic method and devices still need to be developed for the detection of NDV infection. To date, some sensing methods to detect NDV have been put forward. Before using these sensing methods and devices for clinical diagnosis of NDV, their performance should be further evaluated and validated.

With the widespread use of influenza vaccines, surveillance for NDV becomes much more difficult because of the inability to differentiate the infected from vaccinated animals (DIVA). In terms of serology, the antibody level after infection with NDV wild virus and recovery was significantly higher than that after normal vaccine immunization. When the antibody level is abnormally elevated and the antibody uniformity is poor, it is preliminarily considered to have NDV wild virus. But the best way is to immunize with an NDV-marked vaccine or a gene-deleted vaccine. For example, the swine fever rabbit attenuated vaccine is modified so that it lacks a certain gene (such as E0) or a certain segment (such as E2), but its immunogenicity does not change (90, 91). Using the protein expressed by the deleted gene as a diagnostic antigen can distinguish the antibodies produced by vaccinated pigs and naturally infected pigs. Thus, a simple, affordable, reliable, rapid, and diagnostic method and devices still need to be developed for distinguish vaccine-immunized animals from virus-infected animals. In addition, NDV pathogenesis and genetics need to be better understood, facilitating the design of new diagnostic methods and effective drugs and vaccines to counter NDV.

## Author contributions

CW: methodology, resources, and supervision. QM and SM: formal analysis and investigation. QM, SM, PS, PZ, and JW: writing—original draft preparation. QM, CW, and YZ: writing—review and editing. CW, QM, and SL: funding acquisition. All authors read and approved the final manuscript.

## Funding

This research was funded by Xi'an IRIS Livestock Technology Co. Ltd. (K4030218170), China Postdoctoral Science Foundation (2017M610659 and 2018T111113), the State Key Laboratory of Veterinary Etiological Biology, Lanzhou Veterinary Research Institute, Chinese Academy of Agricultural Sciences (SKLVEB2016KFKT014), and the Fundamental Research Funds for the Central Universities (2452019053). The funding bodies played no role in the design of the study and collection, analysis, and interpretation of data and in writing the manuscript.

## Conflict of interest

The authors declare that the research was conducted in the absence of any commercial or financial relationships that could be construed as a potential conflict of interest.

## Publisher's note

All claims expressed in this article are solely those of the authors and do not necessarily represent those of their affiliated organizations, or those of the publisher, the editors and the reviewers. Any product that may be evaluated in this article, or claim that may be made by its manufacturer, is not guaranteed or endorsed by the publisher.
